# Analysis of Clinical Trials on Therapies for Prostate Cancer in Mainland China and Globally from 2010 to 2020

**DOI:** 10.3389/fonc.2021.647110

**Published:** 2021-05-12

**Authors:** Kun Chen, Kehua Jiang, Lannan Tang, Xiaolong Chen, Jianxin Hu, Fa Sun

**Affiliations:** ^1^ NHC Key Laboratory of Pulmonary Immune-Related Diseases, Guizhou Provincial People’s Hospital, Guiyang, China; ^2^ Department of Urology, Guizhou Provincial People’s Hospital, Guiyang, China

**Keywords:** drug trials, prostate cancer, chemotherapy, gene-targeted, immunotherapy

## Abstract

The overall aging of the world population has contributed to the continuous upward trend in the incidence of prostate cancer (PC). Trials on PC therapy have been extensively performed, but no study has analyzed the overall trends and characteristics of these trials, especially for those carried out in China. This study aimed to provide insights on the future direction of drug development in PC, thus supplying essential supportive data for stakeholders, including researchers, patients, investors, clinicians, and pharmaceutical industry. The details of the clinical trials of drug therapies for PC during January 1, 2010, to January 1, 2020, were collected from Pharmaprojects. A total of 463 clinical trials on different therapies with 132 different drugs were completed. The long-acting endocrine therapy with few side effects, radiotherapy combined with immune checkpoint inhibitors, gene-targeted chemotherapeutics, and novel immunotherapeutic products changed the concept of PC treatment. In mainland China, 31 trials with 19 drugs have been completed in the 10 assessment years. China has initiated a few trials investigating a limited number of drug targets, centered in a markedly uneven geographical distribution of leading clinical trial units; hence, the development of PC drugs has a long way to go. Given the large patient pool, China deserves widespread attention for PC drug research and development. These findings might have a significant impact on scientific research and industrial investment.

## Introduction

Prostate cancer (PC) is the most common non-cutaneous malignancy and the second most common cause of cancer-related death among men in the United States. Estimates revealed 191,930 (21%) new cases and 33,330 (10%) cancer-related deaths in 2020 in the United States. The lifetime risk of developing PC is 11.6% (1, 2). The situation in China is of concern in part because of the rapid population growth and socioeconomic development. In 2016, 120,000 new cases of PC were reported in China. China has greatly increased the global cancer burden because of its large population size, which is approximately one-fifth of the world population; almost 22% of new cancer cases and nearly 27% of cancer deaths worldwide occurred in China (3, 4). The increasing average age of the population has contributed to an upward trend in the incidence of PC and related mortality.

Although the 5-year survival rate of localized PC is more than 99%, advanced PC is generally considered incurable (5). The 5-year survival rate of metastatic PC is only 31%. Therefore, effective new drugs and combination therapies are urgently needed. Since the 1940s, the suppression of gonadal production of testosterone *via* androgen deprivation therapy has been the backbone of the management of advanced PC (6). The currently approved agents for treating advanced PC act by either inhibiting the androgen axis (Abiraterone, Enzalutamide, Apalutamide, and Darolutamide), targeting microtubules by inhibiting depolymerization or promoting polymerization (Docetaxel and Cabazitaxel), using radioactive calcium mimetics targeting bone metastases (radium-223, Ra 223), or employing immune-related mechanisms (Sipuleucel-T).

A rapid change in PC treatment is now witnessed with the increased understanding of the evolution, signaling pathways, mutational profiles, and drug resistance mechanisms. However, data analysis of the drug trials for PC and how they evolved over the past decade were lacking. Thus, this systematic review was performed on the trends of drug therapeutic development for PC in China and globally during 2010–2020. The study aimed to provide insights on the future direction of drug development in PC, thus supplying essential supportive data for stakeholders, including researchers, patients, investors, clinicians, and pharmaceutical industry.

## Data Collection and Analysis

### Search Strategy and Selection Criteria

This study comprehensively analyzed all the active or completed clinical trials of drug therapeutics for PC from January, 2010, to January 1, 2020, worldwide and in China. The details of the trials were obtained from Pharmaprojects, a drug development database developed by INFORMA (https://pharma.id.informa.com). Pharmaprojects harbors more than 40,000 public sources, including Clinicaltrials.gov and Gene Therapy Clinical Trials Worldwide, as well as many other sources. The following search keywords were used: [(Actual Start Date is from 2010/01/01 to 2020/01/01) AND (Disease is oncology: Prostate) AND (Drug Disease is cancer, prostate) OR [(Drug Disease is cancer, prostate, neuroendocrine) AND (Development Status is Active) AND (Trial Status is completed)].

The INFORMA platform documented 128,464 trials with 11,931 drugs spanning January 1, 2010 to January 1, 2020. Data processing was divided into four steps. First, all trials based on the disease (oncology: Prostate) were included, and 1,774 trials of PC with 693 drugs were identified. Second, the therapeutic action was chosen as anti-PC, and 1,243 PC drug trials with 343 drugs were included in the following analysis. Many drug trials were not actually carried out due to trial design failures or inclusion difficulties. Therefore, all trials with development status active (included complete) were included. Manual data extraction was not required because this information was already available on the INFORMA database, a leading international research group. With data corrected, 1,041 trials of PC with 230 drugs were identified and used to determine the trends. A total of 463 trials with 132 drugs targeting PC were completed and included in the final analysis. Finally, assignment was done according to the phase, primary endpoint, or therapeutic classification (endocrine therapy, chemotherapy, radiotherapy, and immunotherapy).

### Statistical Analysis

SPSS Statistics 22.0 was used for data analysis. Number (%) was used for qualitative variables. The 10-year trends in the selected indicators were observed, including the number of completed trials, proportion of phases I–IV, trial regions (country), primary endpoint (safety or efficacy), line of therapy, and disease stage. The year of a trial was defined as the start date of the trial.

## Results

### Trends of Clinical Trials on PC Therapy Over Time

**Figure 1:**
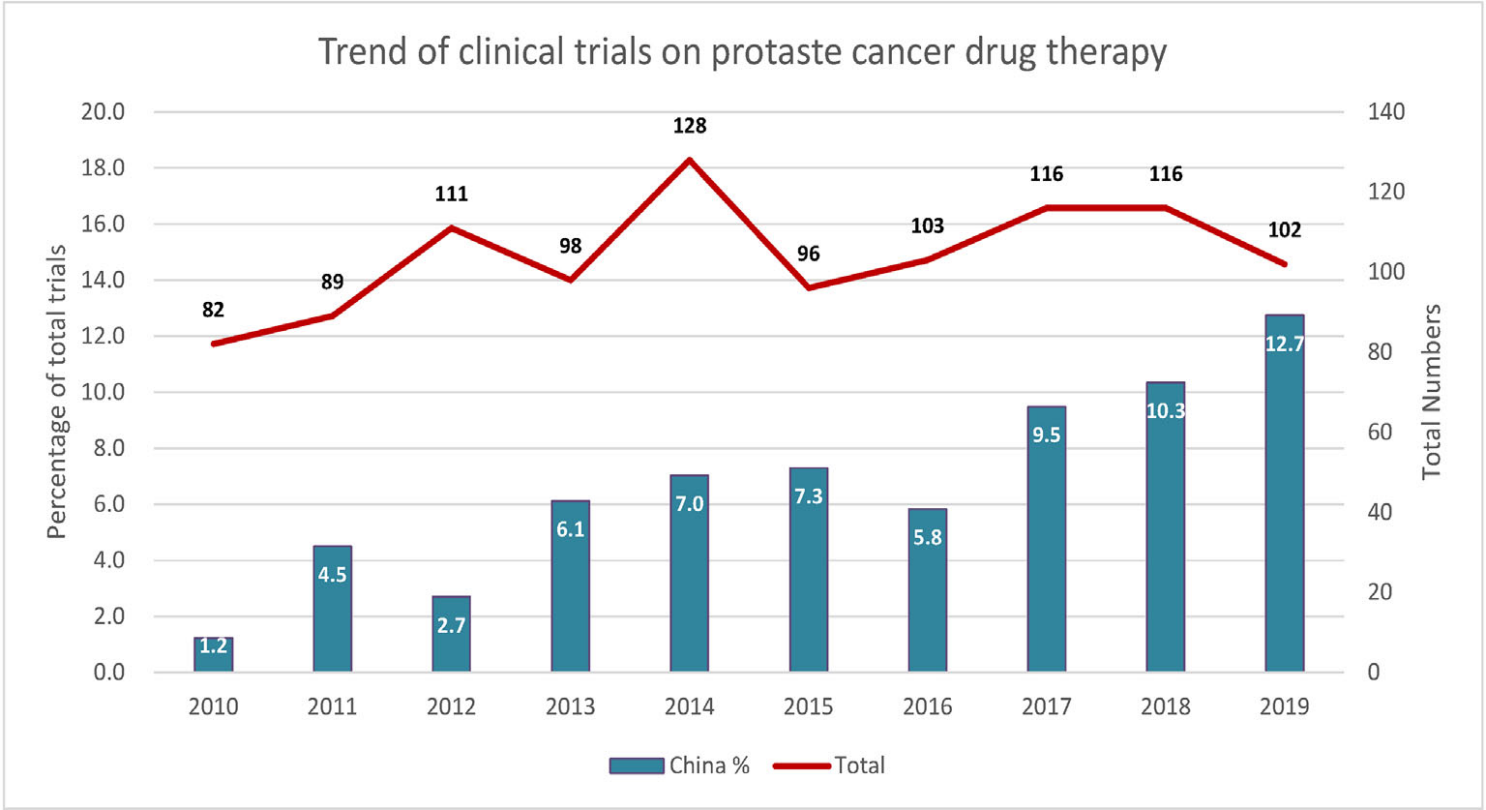
Trends of clinical trials of prostate cancer therapy in China and worldwide. Left *y*-axis indicates the ratio of total trials, and right *y*-axis indicates the total number of trials.

From January 1, 2010 to January 1, 2020, 1041 clinical trials testing 230 PC drugs were carried out worldwide. The total number of trials worldwide has not much changed over the past 10 years with an average annual growth rate of 2.5% (**Figure 1**). However, China represents an increasing percentage of the total trials, especially after 2017, with an average annual growth rate of 29.8%.

### Characteristics of Global Clinical Trials on Drug Therapeutics for PC

Based on a comprehensive review of the published literature and data available from active clinical trials on therapeutic drugs for PC, the drugs were classified into the following treatment classes: chemotherapy, endocrine therapy, immunotherapy, and radiotherapy. A total of 463 clinical trials were completed between 2010 and 2020, including 192 (41.5%) on endocrine therapy, 145 (31.3%) on chemotherapy, 96 (20.7%) on immunotherapy, and 30 (6.5%) on radiotherapy trials (**Table 1**). Most of these trials were phase I or phase II trials (372/463, 80.3%), whereas only 91 were phase III or IV trials (19.7%) (**Table 1**). The early phase clinical trials on immunotherapy and chemotherapy accounted for 94.8 and 87.6% of trials, respectively. The trials were conducted mainly in the United States and Europe, with more than half in the United States (252/463, 54.4%). In the United States, the proportion of endocrine therapy, chemotherapy, and immunotherapy was similar in trials. However, clinical trials involving endocrine therapy were 21 (21/31, 67.7%) in China. Safety was the most common primary endpoint for chemotherapy and immunotherapy trials, and efficacy was the primary endpoint for endocrine therapy and radiotherapy trials. The lines of therapy were based on first line (155/463, 33.5%) and second line (199/463, 43.0%). Endocrine therapy and immunotherapy covered all lines of therapy, including neoadjuvant and adjuvant. Chemotherapy, endocrine therapy, and immunotherapy trials covered early and late stages of prostate cancer. Radiotherapy is generally used for later-stage disease. However, three trials used radiotherapy to treat early stage disease.

**Table 1:** Classification and characteristics of global clinical trials on drug therapeutics for prostate cancer.

Characteristic	Type	Chemotherapy n (%)	Endocrinotherapy n (%)	Immunotherapy n (%)	Radiotherapy n (%)	Totally n (%)
Phase	I	81(55.9)	49(25.5)	55(57.3)	11(36.7)	196(42.3)
	II	46(31.7)	84(43.7)	36(37.5)	10(33.3)	176(38.0)
	III	7(4.8)	29(15.1)	2(2.1)	6(20.0)	44(9.5)
	IV	11(7.6)	30(15.6)	3(3.1)	3(10.0)	47(10.2)
Trial Region/Country	US	79(54.5)	88(46.1)	74(77.1)	11(36.7)	252(54.4)
	China mainland	6(4.1)	21(10.5)	1(1.0)	3(10.0)	31(6.7)
	Europe	62(42.8)	69(36.1)	38(39.6)	16(53.3)	185(40.0)
Primary endpoint	Safety	77(53.1)	59(30.9)	54(56.2)	13(43.3)	203(43.8)
	Efficacy	68(46.9)	133(69.1)	42(43.8)	17(56.7)	260(56.2)
Line of therapy	Neoadjuvant	3(2.1)	17(8.9)	4(4.2)	2(6.7)	25(5.4)
	Adjuvant	0	8(4.2)	5(5.2)	0	13(2.8)
	First line	38(26.2)	64(33.0)	38(39.6)	15(50.0)	155(33.5)
	Second line	96(66.2)	44(23.0)	46(47.9)	13(43.3)	199(43.0)
	Latter line	27(18.6)	10(5.2)	19(19.8)	1(3.3)	57(12.3)
	NA	18(12.4)	73(38.0)	12(12.5)	6(20.0)	109(23.5)
Stage of diseases	Early stage	21(14.5)	36(18.8)	18(18.8)	3(10.0)	79(16.8)
	III/IV	71(49.0)	71(37.0)	49(51.0)	1(3.3)	192(41.5)
	I V	60(41.4)	85(44.3)	38(39.6)	26(86.7)	209(45.4)
	N A	5(3.4)	24(12.5)	0	0	29(6.3)
Totally		145(31.3)	192(41.5)	96(20.7)	30(6.5)	463

Phase: I, I/II, and II are early stage clinical trials.

Trial region/country: The region or country covered by clinical trials.

NA, Not applicable.

### Classification of Drugs in Clinical Investigations

**Figure 2:**
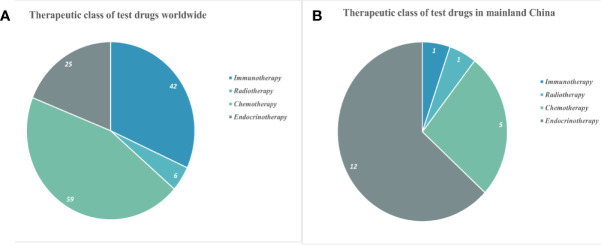
Classification of drugs in clinical trials for prostate cancer worldwide **(A)** and in mainland China **(B)**.

Globally, 132 different drugs were used for treating PC in all completed trials, including 25 (18.9%) endocrine therapy, 59 (44.7%) chemotherapy, 6 (4.6%) radiotherapy, and 42 (31.8%) immunotherapy (**Figure 2**). Trials investigating 19 drugs targeting PC were completed in mainland China in the last 10 years, including 12 (67.7%) for endocrine therapy, 5 (19.4%) for chemotherapy, 1 (9.7%) for radiotherapy, and 1 (3.2%) for immunotherapy (**Figure 2**). The treatments for PC evaluated in trials over the past 10 years were predominantly chemotherapeutic worldwide, whereas endocrine therapy was the dominant therapy in China.

### Analysis of Clinical Trials in Mainland China

**Figure 3:**
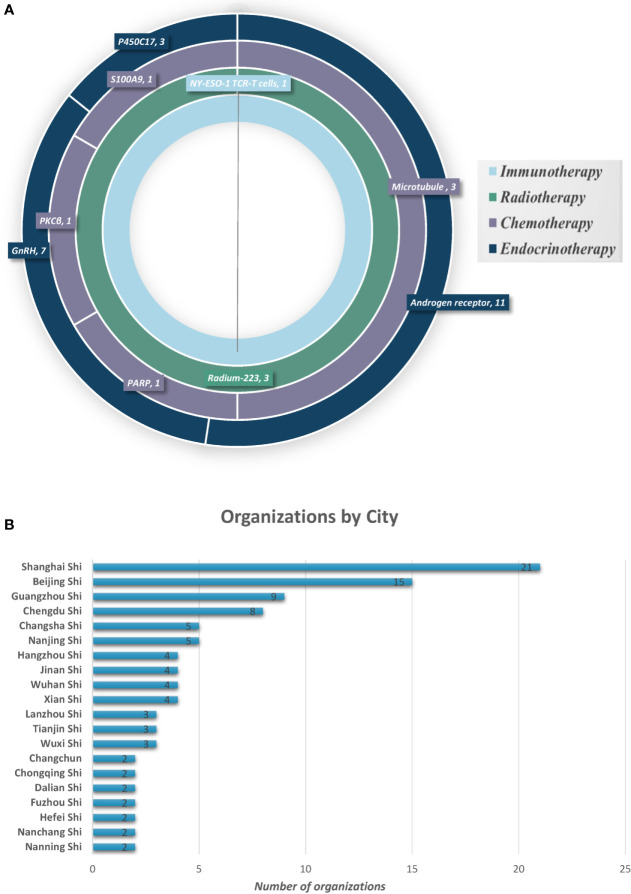
Prostate cancer therapy trial classification and geographical locations. **(A)** Therapy type and targets for PC in clinical trials in mainland China. **(B)** Cities with organizations for PC drug clinical trials (showing top 20).

The clinical investigations on the use of therapeutic drugs for PC in mainland China and worldwide were compared. In mainland China, 31 trials were completed in the last 10 years, including 21 on endocrine therapy, six on chemotherapy, three on radiotherapy, and one on immunotherapy. Most of these trials were phase III or phase IV trials (19/31, 61.3%), whereas only 12 were phase I or II trials (12/31, 38.7%). The therapeutic targets in each of these trials were identified (**Figure 3**). Endocrine therapy targets included androgen receptor (AR) (11/21, 52.4%), gonadotropin-releasing hormone (GnRH) (7/21, 33.3%), and cytochrome p450c17 (3/21, 14.3%). Chemotherapy targets included microtubules (3/6, 50%), poly(ADP-ribose) polymerase (PARP) (1/6, 16.6%), PKCβ (1/6, 16.6%), and S100A9 (MRP-14 or calgranulin B) (1/6, 16.6%). All three radiotherapy trials used radium-223 (Ra 223) to target actively dividing cancer cells (3/3, 100%). The single immunotherapy trial was a T-cell therapy targeting NY-ESO-1. China differs from other regions of the world in that drug targets in mainland China—initiated trials are limited. Moreover, the number of organizations conducting clinical trials on PC therapies is relatively low (125 organizations); these trials were mainly done in large cities such as Shanghai, Beijing, Guangzhou, and Chengdu (**Figure 3**).

## Discussion

**Figure 4:**
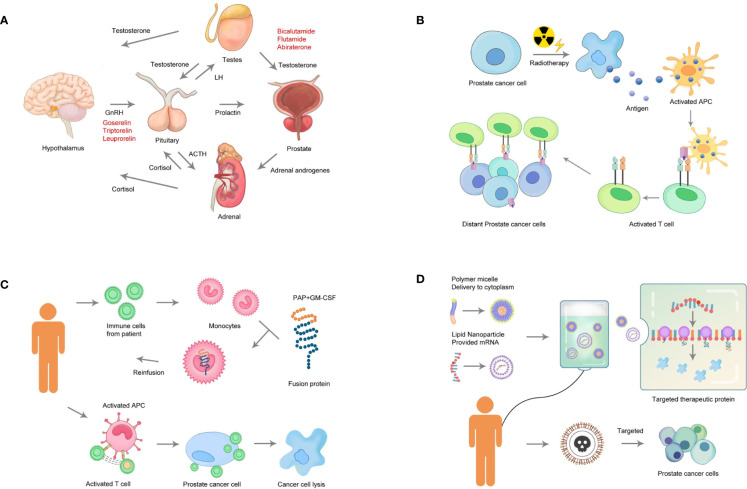
Categories of therapeutic strategies for prostate cancer. **(A)** Endocrine therapy: Androgens stimulate the growth of PC. Drugs to suppress androgen biosynthesis as therapy for PC (androgen depletion) target adrenocorticotropic hormone (ACTH), gonadotropin-releasing hormone (GnRH), and luteinizing hormone (LH). **(B)** Radiotherapy: Radiotherapy directly targets the tumor cells, which is followed by the release of tumor antigens and the activation of immune responses. **(C)** Immunotherapy: The cells are obtained from the patient. The cells highly expressing the target gene or matching the tumor antigen are selected for expansion *in vitro*. Finally, the expanded cells are transplanted back into the patient. **(D)** Chemotherapy: Effective components of a gene-targeted chemotherapeutic drug include a polymer micelle that delivers cytotoxic chemicals and mRNA. Loading of the therapeutic protein can lead to the release of toxic components from the PC cells, resulting in cell apoptosis.

This systematic review provided substantial information on the landscape of drug and therapy development for PC in the last decade. The drugs were classified into the following treatment classes: chemotherapy, endocrine therapy, immunotherapy, and radiotherapy. These four classes were described, and the potential agents were listed (**Figure 4**), providing a critical review on several promising therapeutic agents with novel targets.

### Endocrine Therapy

Currently, an increasing number of clinicians and patients have turned to androgen deprivation therapy as an alternative to surgery, radiation, or conservative management, especially for older men (6). Among such endocrine therapies are GnRH agonists, which are synthetic peptides modeled after the hypothalamic neuro-hormone GnRH, which interacts with the GnRH receptor to elicit its biologic response (**Figure 4**). GnRH antagonists, a similar but distinct strategy, represent a new class of endocrine therapy that induces a more rapid suppression of serum testosterone compared with GnRH receptor agonists, but without the testosterone surge associated with the latter. The principal mechanism of GnRH antagonists is competitive binding to the GnRH receptor (5). In 2008, the GnRH antagonist Degarelix was approved by the United States Food and Drug Administration (FDA) for treating advanced PC (7–9). After 3 years (2011), Degarelix was approved by FDA for the treatment of castration-resistant PC (CRPC) (7). The results of the two pooled analyses suggested that the incidence of musculoskeletal adverse events, including back pain, myalgia, arthralgia, spinal column stenosis, and fracture, was lower in the Degarelix group than in the GnRH agonist group. Another strategy to achieve androgen deprivation is Abiraterone, which targets androgen production by inhibiting cytochrome p450c17, a rate-limiting enzyme in androgen biosynthesis (10). This drug interrupts androgen production at three sources, that is, the testis, the adrenal glands, and the tumor itself. However, the use of abiraterone as a treatment for PC is associated with several undesirable physiological side effects, including a decrease in cortisol levels and a compensatory increase in adrenocorticotropic hormone (ACTH) (10, 11). The antiandrogen Enzalutamide (commercially known as XTANDI) is a small molecule that binds to the AR at the androgen-binding site, thus preventing the transfer of the AR to the nucleus and suppressing any possible tumor agonist-like activity. Additionally, this drug inhibits DNA-binding and co-activator recruitment by the AR, leading to cellular apoptosis and decreased prostate tumor volume. The FDA has approved XTANDI for metastatic CRPC in patients who received previous docetaxel treatment (2012) for the treatment of non-metastatic CRPC (2018) and metastatic castration-sensitive PC (CSPC) (2019) (12–15). In many respects, the ability of endocrine therapy to control PC has overshadowed its toxicity and impact on the quality of life. The endocrine therapy covered all lines of therapy, including neoadjuvant and adjuvant, which also overlapped with the early and later stages of PC. Recently, a mounting body of literature has highlighted the toxicities associated with endocrine therapy, including cardiovascular diseases; harmful metabolic changes such as obesity, insulin resistance, and diabetes; dyslipidemia; and a higher risk of bone fracture (16, 17). Thus, more effective, specific, and well-tolerated agents are needed, which can provide a longer and better quality of life while ameliorating the side effects related to disease and treatment morbidity.

### Radiotherapy

Two major categories of radiotherapy for PC are external beam radiotherapy (EBRT) and brachytherapy (BT), both of which have improved with the use of high-quality multimodal imaging and robotic assistance (18, 19). A large number of randomized controlled clinical trials have assessed the efficacy and safety of radiotherapy (20, 21). Ra-223 is a first-in-class, *α*-particle-emitting, radioactive agent used as the first-line therapy for PC (FDA approved in 2013), which has provided an additional treatment option for patients with CRPC and symptomatic bone metastases in the absence of visceral metastases (22). The safety and tolerability of Ra-223 for metastatic CRPC have been demonstrated in patients administered up to six intravenous injections (23). Strontium-89 (Sr-89), in combination with EBRT, increased the durability of pain relief in patients with multiple bone metastases, and the addition of Sr-89 to the chemotherapy of similar patients resulted in a small increase in quality-adjusted life-years (24). As a monotherapy for patients with multiple bone metastases from CRPC, Sr-89 effectively decreased pain at the cost of mild-to-moderate myelosuppression (25, 26) and increased the duration of response when combined with either EBRT or chemotherapy (26, 27). Other radiopharmaceuticals, such as rhenium-188 and rhenium-186, are still under investigation and hence not yet available for widespread use. The complications of radiotherapy in treating PC include the development of impotency, lower urinary tract symptoms, and hematuria. Hematospermia, orgasmalgia, and alteration in the intensity of orgasm of limited duration were documented in 26, 15, and 38% of cases, respectively. Treatment differences were observed especially in sexual, urinary, and bowel functions. The bowel function was worse after EBRT compared with radical prostatectomy. Radiotherapy is typically used to treat late-stage PC; however, three trials tested radiotherapy for early stage PC (NCT01851018, NCT01875393, and NCT01310894). Thus, more attention should be paid to the toxic and side effects of radiotherapy to provide better life experience for patients. Radiotherapy combined with immune checkpoint inhibitors (ICI) is currently under investigation as a treatment strategy for PC (**Figure 4**). Many clinical trials characterized the toxicity of ICI plus radiotherapy compared with ICI alone. The results showed that the toxicities of ICI plus radiotherapy and ICI alone were similar (28–31). Thus, it appeared that ICI plus radiotherapy was a feasible treatment option and warranted further exploration.

### Immunotherapy

Immunotherapy uses a person’s own immune system to fight cancer, including chimeric antigen receptors, T-cell receptor-engineered T-cell therapy, dendritic cell vaccines, DNA vaccines, oncolytic virus vaccines, traditional antibody drugs, and so forth. In the last 10 years, early phase, immunotherapy clinical trials accounted for 94.8% of all the therapeutic trials on PC, indicating that this was a major new focus in PC therapy development. In 2010, the first dendritic cell vaccine, Sipuleucel-T, was approved by the FDA for metastatic, asymptomatic, hormone-refractory PC in patients with a 3-year survival rate of 34% (32) (**Figure 4**). This opened a new chapter in immunotherapy for PC. Chimpanzee adenovirus and modified vaccinia Ankara (ChAd-MVA) 5T4 vaccine, a recombinant virus encoding human tumor-associated antigen 5T4, promotes the immune system killing of cancer cells that express 5T4 on their surface. This strategy is being evaluated as a neoadjuvant therapy, and 40 patients have been enrolled (NCT02390063). MVI-118 (pTVG-AR) is an intradermal prostatic acid phosphatase encoding DNA plasmid gene–based immunotherapy that has completed phase II clinical trials and enrolled 99 patients with adenocarcinoma of the prostate (NCT01341652). The use of tumor-infiltrating lymphocytes grown *ex vivo* from resected cancer tissue and reapplied to the patient *via* an intravenous infusion (adoptive cell therapy) has completed phase I/II clinical trials with 25 patients with tumor enrolled (NCT03296137). TAEST-16001 (NY-ESO-1 T cells) is a specific T-cell receptor–based immunotherapy product and is the only cell therapy that has completed clinical trial for treating solid tumors in China. In the last few years, immunotherapy has become an important cancer treatment modality. However, it is unlikely that any of the immunotherapeutics alone can dramatically change PC outcomes, but combination strategies are more promising and provide a reason for optimism. Several completed and ongoing studies have shown that the combination of cancer vaccines or checkpoint inhibitors with different immunotherapeutic agents, hormonal therapy (Enzalutamide), radiotherapy (Radium 223), DNA-damaging agents (Olaparib), or chemotherapy (Docetaxel) can enhance immune responses and induce more dramatic, long-lasting clinical responses without significant toxicity. The goal of PC immunotherapy does not have to be the complete eradication of advanced disease but rather the return to an immunologic equilibrium with an indolent disease state. Besides determining the optimal combination of treatment regimens, efforts are also ongoing to discover the biomarkers of immune response. With such concerted efforts, the future of immunotherapy in PC looks brighter than ever.

### Chemotherapy

Before the United States FDA approval of Docetaxel in 2004 for the treatment of metastatic CRPC (33), no standard second-line chemotherapy was available to improve overall survival. Another tubulin-binding taxane drug, Cabazitaxel, was also FDA approved to treat PC as second-line salvage chemotherapy in 2010 (34). However, Docetaxel failed as the first-line chemotherapy, and Cabazitaxel also showed more adverse effects. Thirty-eight trials on first-line chemotherapy have been completed in the last decade, and diverse combinations have been attempted but not found to be successful (35). Recently, interest has grown in neoadjuvant treatment to improve surgical outcomes. Three clinical trials on chemotherapy neoadjuvant were completed during 2010–2020. One such treatment was nanoparticle paclitaxel that completed phase II clinical trial with 16 patients enrolled diagnosed with PC and scheduled for prostatectomy (NCT03077659). Nanoparticle paclitaxel suspensions are administered by intratumoral injection directly to the site of a tumor, where they become entrapped and continuously release drug molecules as they dissolve. With the development of gene editing technology, promising therapeutic agents using this novel strategy are available, for example, IGF–MTX, an insulin-like growth factor (IGF) conjugated to chemically engineered methotrexate (MTX). This drug has completed phase I clinical trials, with 92 patients enrolled with tumors expressing IGF-1R (NCT02045368). IGF–MTX targets IGF receptor proteins on cancer cells to precisely deliver the drug. The principle of gene-targeted chemotherapy is to release toxic components to the PC, with healthy cells receiving very less toxicant (**Figure 4**). Gene-targeted chemotherapy holds great potential for PC.

Moreover, most trials were performed and designed to improve treatments for non-metastatic PC (67%). More than half of all trials identified tested the effects of systemic treatment, with some in patients with non-metastatic disease (36, 37). However, patients with metastatic castration-resistant disease require more aggressive systemic therapy. Thus, improvements in systemic treatments, even in the non-metastatic setting, are very important and may positively impact cancer control rates. Trials focused on not only local treatment methods, such as radiation therapy or immunotherapy, but also surgery for PC. The study analysis demonstrated strong ongoing clinical research activity, which might undoubtedly impact the future of PC treatment worldwide.

This study revealed that the number of trials on PC therapies has not much changed over the past 10 years worldwide, with an average annual growth rate of 2.5%. Even then, China represents an increasing percentage of the total trials globally with an average annual growth rate of 29.8%. The increasing average age of the population has contributed to a clear upward trend in the incidence of PC and related mortality. In 2016, 120,000 new cases of PC were reported in mainland China. It is expected that the number of new cases will reach 237,000 by 2030, with a compound annual growth rate of 5%. The Chinese market for PC drugs is expected to reach $4.8 billion by 2030. China deserves widespread attention in terms of PC drug research and development.

Most of these trials were phase I or phase II trials (372/463, 80.3%), whereas only 91 were phase III or IV trials (19.7%) worldwide. In mainland China, most of these trials were phase III or phase IV trials, whereas only 12 were phase I or II trials (12/31, 38.7%). Phase I and II are direct evidence of innovation. Only if they are successful will the trials proceed to phase III or IV. The inadequacy of phase I or II in China was due to the lack of capability of independent innovation. Hence, China still needs to improve the ability to develop therapeutic drugs for PC. The present review found that phase I or II clinical trials on immunotherapy and chemotherapy accounted for >85% of all trials globally, indicating that many new trials were being conducted and novel drugs were being developed based mainly on these two aspects. The United States has completed more than half of the clinical trials (252/463, 54.4%); the proportion of treatment types in endocrine therapy, chemotherapy, and immunotherapy was similar. Endocrine therapy has been the backbone of the management of advanced PC. However, the majority of the novel drugs being developed are based on immunotherapy and chemotherapy. This uniformity represents the balance between tradition and innovation. In contrast, China has completed only 31 (31/463, 6.7%) clinical trials, mainly involving endocrine therapy, and only one trial on immunotherapy. The development of immunotherapy and chemotherapy can not only provide more selectivity for treatment but also increase the possibility of developing new drugs for PC. Additionally, only nine drug targets have been reported in China-initiated trials, with chemotherapy targets including microtubules, PARP, PKCβ, and S100A9, and the immunotherapy target NY-ESO-1. Therefore, China differs from other regions in the limited number of drug targets and trials, particularly compared with the United States. However, the clinical trial centers conducting studies on PC drugs are limited and mainly concentrated in the large cities of Shanghai, Beijing, Guangzhou, and Chengdu. This review further showed a markedly uneven geographical distribution of the leading clinical trial units across China. Therefore, not only uneven population and patient distribution but also geographical disparity are the direct manifestations of the uneven distribution of superior medical resources for clinical research across China (38). How to improve the balance between equitable access to new drugs and the efficiency of pharmaceutical research and development is an important topic worthy of exploration by policymakers.

## Conclusions

The research and development of PC drugs showed rapid progress over the decade 2010–2020. Long-acting endocrine therapy with few side effects, radiotherapy combined with ICI, gene-targeted chemotherapeutics, and novel immunotherapeutic products may change the concept of PC treatment. The clinical investigations in mainland China are limited in terms of the relatively low numbers of drug targets and trials and the markedly uneven geographical distribution of the leading clinical trial centers. Hence, the development of PC drugs has a long way to go. However, given the large patient pool, China deserves widespread attention regarding PC drug research and development. These findings might have a significant impact on scientific research and industrial investment.

## Data Availability Statement

The original contributions presented in the study are included in the article/supplementary material. Further inquiries can be directed to the corresponding author.

## Author Contributions

All authors listed have made a substantial, direct, and intellectual contribution to the work and approved it for publication.

## Funding

This study was funded by Guizhou Provincial Science and Technology Foundation (Number: [2020]4Y142), Guizhou Provincial Science and Technology Foundation (Number: [2020]1Y303), and Foundation of Health and Family Planning Commission of Guizhou Province (Number: gzwjkj2019-1-127). The funding agencies and donors have no role in any aspect of this study. NHC Key Laboratory of Pulmonary Immune-related Diseases supported by the Non-profit central research Institute fund of Chinese Academy of Medical Sciences 2019PT320003.

## Conflict of Interest

The authors declare that the research was conducted in the absence of any commercial or financial relationships that could be construed as a potential conflict of interest.
